# Calendar

**DOI:** 10.1155/S0962935196000130

**Published:** 1996-02


					Calendar
Calendar
1996
18-19 April 1996
New Phospholipases and Cyclooxygenases
as Novel Drug Targets,
Institut Pasteur, Paris, France.
Contact: M.L. Drye, Institut Pasteur
Euroconferences, 28 rue du Docteur
Roux, 75015 Paris, France.
Fax: (+33) (1) 40 61 34 05.
21-26 April 1996
8th APLAR Congress of Rheumatology,
Melbourne, Australia.
Contact: 8th Asia Pacific League
Against Rheumatism Congress,
PO Box 29, Parkxdlle,
3052 Victoria, Australia.
Fax: (+61) (3) 387 3120.
16 May 1996
Apoptosis- Insight into its Role in
Inflammation,
New York Academy of Sciences, USA.
Contact: Dr B.M. Weichman, IRA,
Wyeth-Ayerst Research, CN 8000,
Princeton, NJ 08543, USA.
Fax: (+ 1) 908 274 4738.
20-23 May 1996
United States FDA Medical
Device Update: Design Controls,
GMP Requirements and
Marketing Clearance,
Pails, France.
Contact: Zena Barrick,
Medical Device Technology,
Advanstar House, Park West,
Sealand Road, Chester CH1 4RN, UK.
Fax: (+ 44) (0) 1244 370 011.
31 May-3 June 1996
26th Scandinavian Congress
of Rheumatology,
Reykjavik, Iceland.
Contact: Travel and Congress Bureau,
Feroaskrifstofa Islands,
Iceland Tourist Bureau, Skogarhlio 18,
101 Reykjavik, Iceland.
Fax: (+ 354) 625895/5625895.
4-7 September 1996
2nd International Congress on
Phagocytes: Biology, Physiology,
Pathology and Pharmacotherapeutics,
Pavia, Italy.
Contact: Prof. Giovanni Ricevuti,
Institute of Medical Therapy,
OSM 27100, Pavia, Italy.
Fax: (+ 39) 382 526341.
22-27 September 1996
ICP '96
10th International Conference
on Postaglandins and
Related Compounds,
Vienna, Austria.
Contact: ICOS Congress
Organisation Service Ges.m.b.H.,
Johannesgasse 14
A-1010 Vienna, Austria.
Fax: (+ 43) (1) 512 80 91 80.
27-31 October 1996
New Drugs for Inflammatory,
Allergic, and Immunologic Disease:
A Symposium featured at the
8th Intemational Conference on
Inflammation Research,
Hershey, PA, USA.
Contact: Dr JB Summers,
Abbot Laboratories,
100 Abbott Park Road,
D47J AP10, Abbott Park,
IL 60064, USA.
Fax: (+ 1) 708 938 5034.
1997
16-20 November 1997
The 3rd Wodd Congress
on Inflammation,
Tokyo, Japan.
Contact: The Secretariat,
c/o PMSI Japan Ltd,
Royal Bldg 12-8 Nibancho,
Chiyoda-ku, Tokyo 102, Japan.
Fax: (+ 81) 3 5275 6994.
NOTICE FOR ADVERTISERS
To advertise in this, or any other journal from
Rapid Science Publishers
please contact Helen Dixon
Rapid Science Publishers
2-6 Boundary Row
London SE1 8HN, UK
Tel: (+ 44) (0)171 865 0198; Fax: (+ 44) (0)171 410 6600
Other services available include:
Reprints of key papers
Symposium abstracts and proceedings
Proceedings and review supplements
(C) 1996 Rapid Science Publishers Mediators of Inflammation Vol 5 1996 75

				

